# Temporal irregularity quantification and mapping of optical action potentials using wave morphology similarity

**DOI:** 10.1016/j.pbiomolbio.2019.12.004

**Published:** 2020-11

**Authors:** Christopher O’Shea, James Winter, Andrew P. Holmes, Daniel M. Johnson, Joao N. Correia, Paulus Kirchhof, Larissa Fabritz, Kashif Rajpoot, Davor Pavlovic

**Affiliations:** a Institute of Cardiovascular Sciences, University of Birmingham, UK; b EPSRC Centre for Doctoral Training in Physical Sciences for Health, School of Chemistry, University of Birmingham, UK; c School of Computer Science, University of Birmingham, Birmingham, B15 2TT, UK; d Institute of Clinical Sciences, University of Birmingham, UK; e Institute of Microbiology and Infection, School of Biosciences, University of Birmingham, UK; f Department of Cardiology, UHB NHS Foundation Trust, Birmingham, UK; g Cardiology Specialty, SWBH NHS Trust, Birmingham, UK

**Keywords:** Optical mapping, Wave similarity, Regularity index, Temporal stability, Optical action potential, Atrial Fibrillation, AF, Ventricular Fibrillation, VF, Action Potential, AP, Action Potential Duration, APD, Regularity Index, RI, Optical Wave Similarity, OWS, Optical Action Potential, OAP, Sympathetic Nervous Stimulation, SNS, Pacing Cycle Length, PCL, Premature Atrial Activity, PAA

## Abstract

**Background:**

Cardiac optical mapping enables direct and high spatio-temporal resolution recording of action potential (AP) morphology. Temporal alterations in AP morphology are both predictive and consequent of arrhythmia. Here we sought to test if methods that quantify regularity of recorded waveforms could be applied to detect and quantify periods of temporal instability in optical mapping datasets in a semi-automated, user-unbiased manner.

**Methods and results:**

We developed, tested and applied algorithms to quantify optical wave similarity (OWS) to study morphological temporal similarity of optically recorded APs. Unlike other measures (e.g. alternans ratio, beat-to-beat variability, arrhythmia scoring), the quantification of OWS is achieved without a restrictive definition of specific signal points/features and is instead derived by analysing the complete morphology from the entire AP waveform.

Using model datasets, we validated the ability of OWS to measure changes in AP morphology, and tested OWS mapping in guinea pig hearts and mouse atria. OWS successfully detected and measured alterations in temporal regularity in response to several proarrhythmic stimuli, including alterations in pacing frequency, premature contractions, alternans and ventricular fibrillation.

**Conclusion:**

OWS mapping provides an effective measure of temporal regularity that can be applied to optical datasets to detect and quantify temporal alterations in action potential morphology. This methodology provides a new metric for arrhythmia inducibility and scoring in optical mapping datasets.

## 1 Introduction

Cardiac arrhythmias, such as ventricular and atrial fibrillation (VF/AF), are characterised by complex spatiotemporal activation and repolarisation dynamics (Moe and Abildskov, 1959; Ten Tusscher et al., 2007; Pandit and Jalife, 2013). Advances in knowledge of the mechanistic drivers or origins of these events (Atienza et al., 2012; Schotten et al., 2011) could be used to develop more effective therapies, for example, leading to improvements in radiofrequency ablation procedures (Ravelli and Mase, 2014; Li et al., 2017). However, recordings of cardiac electrical activity in the time preceding and during an arrhythmia are often temporally disorganised and/or irregular. In the case of electrogram recordings, such signals are termed ‘fractionated’ and the more complex morphology reflects local differences in activation, repolarisation and/or fibrotic areas within the tissue, leading to alterations in the amplitude and temporal regularity of the recorded signal (Caldwell and Redfearn, 2012; Baher et al., 2019). In optical mapping, where action potentials (APs) are recorded using voltage sensitive fluorescent indicators, arrhythmias are associated with similar alterations in signal amplitude and temporal heterogeneity (Laughner et al., 2012). For these reasons, signal parameters such as cycle length and AP duration (APD) become inconsistent and hard to define/measure. Thus, novel temporal heterogeneity measures not reliant on defined waveform points/features consisting of repetitive periods of depolarisation and repolarisation are required.

For both electrogram and optical mapping datasets, strategies have been proposed for quantification of electrophysiological properties in the time prior to and during an arrhythmia. Beat to beat analyses have been successfully utilised in several optical mapping studies to highlight temporal instability (Wang et al., 2014; Winter et al., 2018; O’Shea et al., 2019a). These approaches however can be labour intensive, while high throughput methodologies rely on the automated identification of specific signal points, features (e.g. activation and repolarisation times) and phenomena (e.g. alternans) (O’Shea et al., 2019a; Fabritz et al., 2003). These features can become challenging to algorithmically and accurately measure when signal amplitude decreases, as is observed at fast pacing frequencies and during polymorphic arrhythmia (Laughner et al., 2012; O’Shea et al., 2019a). Furthermore, dominant frequency (Laughner et al., 2012) and phase analysis (Umapathy et al., 2010; Kirchhof et al., 1998) have been employed to map regions of the heart that may act as drivers of arrhythmia (e.g. mother rotors) and to predict termination of arrhythmia episodes. However, these methodologies either forgo ‘direct’ analysis of the signal waveforms or rely on manual inspection. Instead, they use mathematical techniques to transform the signal into another form, for example, into the frequency domain via the fast Fourier transformation.

In electrogram-based electrophysiological mapping, the ‘regularity index (RI)’ has been shown to be an effective method to quantify temporal organisation directly (Faes et al., 2002; Ravelli et al., 2005). This method, as first set out in 2002 for atrial electrograms (Faes et al., 2002), computes the similarity between local activation waves with the hypothesis that waves with a lower index are representative of tissue areas with dyssynchronous activation and repolarisation. RI and similar methods have the advantage of quantifying temporal regularity using simple signal processing methods across the entire waveform morphology (Baher et al., 2019; Faes et al., 2002). The RI approach relies on the setting of a threshold value for ‘similarity’. The use of such an approach for analysis of optical signals has not been evaluated, in particular the effects of setting a similarity threshold.

In this study, we have developed and utilised RI mapping in optical mapping datasets, and further develop an analogous but updated approach for use in optical mapping datasets which we term optical wave similarity (OWS). RI and OWS metrics are compared. OWS is measured from optical mapping datasets to demonstrate its utility in detecting and quantifying temporal homogeneity/regularity of model and optical action potentials (OAPs). Temporal heterogeneity is induced by changes in pacing cycle length and sympathetic nervous stimulation (SNS) in guinea pig ventricles and isolated mouse left atria. Robust measures of temporal heterogeneity across the myocardium, recorded using optical mapping methodologies and integrated into freely available software, will potentially help researchers identify novel drivers of arrhythmogenesis (Baher et al., 2019; Winter et al., 2018; Johnson et al., 2010).

## 2 Methods

### 2.1 Animal welfare

All animal procedures were undertaken in accordance with ethical guidelines set out by the UK Animals (Scientific Procedures) Act 1986 and Directive 2010/63/EU of the European Parliament on the protection of animals used for scientific purposes. Experiments were approved by the home office (mouse: PPL 30/2967 and PFDAAF77F, guinea pig: PPL PF75E5F7F) and the institutional review boards at University of Birmingham (mouse) and King’s College London (guinea pig).

### 2.2 Optical mapping

All data utilised in this study were acquired previously, full details of experimental procedures can be found in the relevant publications (Winter et al., 2018; Holmes et al., 2016; Yu et al., 2014).

Briefly, *ex vivo* isolated intact guinea pig whole hearts were imaged utilising an innervated preparation (Ng et al., 2001). Hearts were loaded with voltage dye Di-8-ANEPPS (1 mg/ml in DMSO, 200–300 μl), paced via silver bipolar electrodes and imaged at 0.5  kHz at 64 × 64 pixel resolution (320 μm pixel width). Sympathetic nervous stimulation (SNS) was achieved by bi-lateral stimulation of efferent sympathetic nerves by a decapolar 5-French catheter in the spinal column. Hearts were paced at 170 ms pacing cycle length (PCL), and PCL reduced by 10 ms every 10 beats until induction of ventricular fibrillation (Winter et al., 2018).

Isolated *ex vivo* mouse hearts were loaded with Di-4-ANEPPS (0.125 mg/ml in DMSO, 1 ml), left atria isolated, pinned down in the superfusion chamber and paced using platinum bipolar electrodes and imaged at 0.987 kHz with maximal resolution of 200 × 2048 pixels (Holmes et al., 2016) (71 μm pixel width). Atria were paced at 150 ms PCL, and PCL was reduced by 10 ms every 20 stimuli down to 50 ms. Some atria were also paced at 150 ms PCL for 1 min to observe instances of spontaneous activity, such as premature atrial activity (PAA).

In both sets of experiments the electromechanical un-coupler blebbistatin (15 μM for guinea pig, 42.75 μM for mouse atria) was used to prevent movement of the tissue and motion artefacts on the recordings.

### 2.3 Optical data pre-processing

Spatial filtering of the optical mapping images was performed via a 3 × 3 pixel Gaussian filter. Baseline correction was performed using a top-hat kernel (200 ms length for guinea pig data, 100 ms for mouse) (Yu et al., 2017). No temporal filtering was applied.

**Fig. 1:**
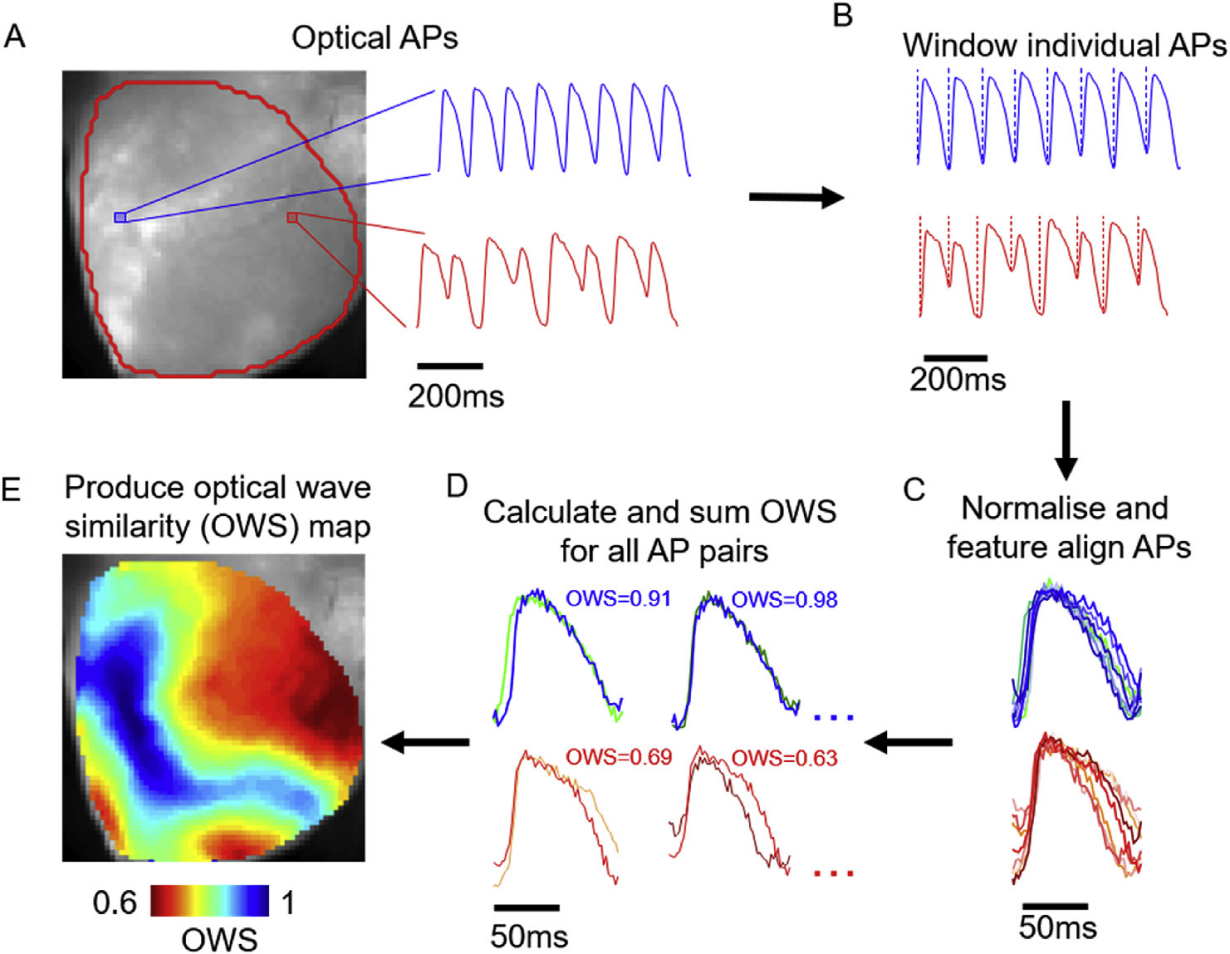
Calculation of optical wave similarity (OWS) from voltage optical mapping data. A) Fluorescence image of voltage dye loaded guinea pig whole heart. Representative signals are shown from the locations marked in blue and red on the fluorescence image. B) Windowing of optical signals based on signal minima. C) Normalisation and alignment of individual optical action potentials (OAPs) in the recorded optical signal. D) Example calculations of OWS from OAP pairs. E) Constructed map of OWS following analysis steps set out in A-D.

Image stacks were then segmented based on PCL. PCL segmentation of the image stack was performed based on the tissue average signal, $F_{tissue}\left(t\right),$

1 $$F_{tissue}\left(t\right)=\overset{N}{\underset{n=1}{\sum }}f_{n}\left(t\right)$$

where $f_{n}\left(t\right)$ is the fluorescent value of pixel n at time t. Only the N pixels which were selected following thresholding (i.e. pixels inside the red outline in Fig. 1A) were included for summation in equation (1) and all subsequent analysis. Peaks in $F_{tissue}\left(t\right)$ were then identified by setting a threshold amplitude (half of maximum $F_{tissue}\left(t\right)$) and minimum peak distance (40 ms for both guinea pig and mouse). The PCL was then defined as the time period between one peak and the previous. The signal was then segmented when changes in PCL of 10 ms were identified. During ventricular fibrillation (VF), automatic PCL based segmentation was not used due to variable time intervals and lower signal amplitude. Instead, a custom selection of time period where the tissue averaged signal clearly demonstrated VF was performed (O’Shea et al., 2019a).

### 2.4 Computationally modelled action potentials

Alongside application of OWS to optically mapped datasets, mathematical models of guinea pig (Luo and Rudy, 1994) and mouse (Bondarenko, 2004) ventricular action potentials were used to validate use of OWS for AP waveforms. Temporal alterations in the action potential morphology were induced by altering the conductance of the respective repolarising potassium channels in each model for odd beats only. This induced alternans like behaviour in the models. For the guinea pig action potentials, potassium channel (IK and IK_1_) conductance was increased by 70%, while for the mouse potassium channel (I_to_, IK_ss_, IK_s_, IK_1_, IK_ur_) conductance was decreased by 45% (Clerx et al., 2016). 1000 beats were simulated in total to allow the models to reach steady-state, and the last 20 beats used for analysis (see below).

The model traces were down sampled to match the recording rates of the optical mapping systems used in this study (0.5 kHz for guinea pig, 1 kHz for mouse). To study the effects of signal quality, noise was added to the datasets by altering the AP by a value selected from a set of normally distributed pseudorandom numbers, the standard deviation of which was set as a percentage value of the action potential amplitude. Due to its pseudorandom nature, this operation was repeated 15 times for each noise level tested. Note: For direct comparison to noise levels of experimental recording, optical noise values were retrieved in the inverse manner, i.e. the amplitude of the optical action potential (OAP) was compared to the standard deviation of the signal at diastolic baseline. These studies were performed using the myokit software (http://myokit.org) (Clerx et al., 2016) and MATLAB (The MathWorks).

### 2.5 Optical wave similarity

#### 2.5.1 Windowing

Optical wave similarity (OWS) and regularity index (RI) were calculated via adaptation of the method set out by Faes et al. for use in bipolar electrograms (Faes et al., 2002). Following the PCL detection, signals were time windowed, Fig. 1B. Unless stated otherwise, for guinea pig action potentials, window timeframe was set at 50 ms before and 150 ms after the peak in the tissue averaged signal ($F_{tissue}\left(t\right),$equation (1)). Due to the shortening of the diastolic interval at faster PCLs (Shattock et al., 2017), the signals were further windowed by the minimum before and after each peak before OWS calculation. The time window used for measuring the OWS between two OAPs was from the closest minima (of either OAP) before and after the peak in the signals. Due to the shorter OAP morphology and smaller tissue area of murine atria (Riley et al., 2012), a constant windowing timeframe of 20 ms before and 40 ms after the peak was utilised for these recordings and no further windowing based on signal minima was required.

#### 2.5.2 Normalisation and signal alignment

Following PCL detection, segmentation and windowing of individual OAPs, the image stack was split into sections (up to 10 sections of 10 beats for guinea pig hearts, 7 sections of 20 beats for mouse atria) matching the PCL protocols described above. For single beat OWS analysis, the individual OAP was compared with the subsequent OAP. Signals were aligned according to the peak times in tissue average signal, equation (1).

For the computationally modelled datasets, OWS analysis was applied to the final 20 beats of 1000 stimulations, to ensure the models had reached steady state. The model APs were aligned by the peak.

#### 2.5.3 Calculation of wave similarity

To calculate wave similarity, we utilised the cosine similarity metric. First, the individual, windowed OAPs (A) were zero corrected (translated so the mean value was zero) and normalised as shown in equation (2),

2 $$A_{n}=\frac{A}{\sqrt{{\sum_{k=1}^{K}A\left(k\right)}^{2}}}$$

where $A_{n}$ is the normalised signal and K is the number of samples in the OAP after windowing, Fig. 1C. The wave similarity, s, between two successive normalised and aligned OAPs, $A_{1}$ and $A_{2}$, was then calculated as,

3 $$s\left(A_{1},A_{2}\right)=\left(A_{1}.A_{2}\right)$$

where $A_{1}.A_{2}$ denotes the scalar dot product (Fig. 1D). The result of equation (3) is a value approaching 1 when $A_{1}$ and $A_{2}$ exhibit a similar morphology, 0 when the two OAPs are morphologically distinct (e.g. two signals of just noise), and −1 if the signals are identical but of opposite phase. For a sequence of OAPs from each pixel, the overall OWS was then calculated from all paired wave similarity values as,

4 $$OWS=\frac{2}{M\left(M-1\right)}\sum_{i=1}^{M}\sum_{j=i+1}^{M}s\left(A_{i},A_{j}\right)$$

where M is the number of OAPs in the sequence. As previously stated, when undertaking single beat OWS analysis, an individual OAP was compared with the subsequent OAP.

To calculate regularity index (RI), the method of Faes et al. originally applied to atrial electrograms was employed (Faes et al., 2002). The wave distance, d, between two normalised and aligned OAPs, $A_{1}$ and $A_{2}$ was calculated as,

(5) $$d\left(A_{1},A_{2}\right)=arccos\left(A_{1}.A_{2}\right)$$

where arccos is the inverse cosine function.

A threshold distance, ε, was then set, and RI of a train of M OAPs measured as

(6) $$RI=\frac{2}{M\left(M-1\right)}\overset{M}{\underset{i=1}{\sum }}\overset{M}{\underset{j=i+1}{\sum }}H(\varepsilon -d\left(A_{i},A_{j}\right))$$

where H is a Heaviside step function which equals 1 when $d\left(A_{i},A_{j}\right)\leq \varepsilon$ (i.e. when two OAPs are similar) and 0 when $d\left(A_{i},A_{j}\right)>\varepsilon$ (i.e. when two OAPs are dissimilar). By the definitions in equations 4, (6)), both higher OWS and RI indicate a greater temporal regularity.

### 2.6 Alternative metrics

To compare the results of OWS analysis with other methodologies, we also conducted APD alternans and dominant frequency analysis.

#### 2.6.1 APD alternans (*ΔAPD*_*80*_)

APD_80_ was calculated as the interval between the maximum upstroke velocity during depolarisation (dF/dt_max_) and the time of 80% repolarisation to baseline. APD_80_ alternans magnitude (*ΔAPD*_*80*_) was then calculated as the absolute difference between APD_80_ of one OAP compared to the previous at each location in the image (Winter et al., 2018; O’Shea et al., 2019b). In some cases, especially at short PCLs, 80% repolarisation was not observed before the next depolarisation. These areas were excluded from analysis (see blue areas in Fig. 6Aii). The results were then averaged for each PCL.

#### 2.6.2 Dominant frequency analysis

For dominant frequency mapping, the frequency spectrum of the signal was computed using fast Fourier transform, with a Hann window applied. Zero padding was applied to achieve a frequency resolution of 0.05 Hz, and the frequency range studied was from 0.5 to 50 Hz (Li et al., 2017).

### 2.7 Statistical analysis and data availability

Unless stated, data are presented as mean ± standard error of the mean. As all interventions are paired in the same samples, group mean differences were tested via two-tailed paired student’s t-test. Significance was defined as P < 0.05.

The pre-processing and signal segmentation described here, as well as all subsequent steps to calculate wave similarity and regularity index have been integrated into an updated version of our electrophysiological mapping software ElectroMap (O’Shea et al., 2019a) (https://github.com/CXO531/ElectroMap).

## 3 Results

### 3.1 Principles of wave similarity measurements

The steps for calculating OWS from optically recorded samples are illustrated in Fig. 1. The key steps in OWS analysis from optically recorded signals (Fig. 1A) are windowing (Fig. 1B), normalisation (equation (2)), feature alignment (Fig. 1C) and OWS calculation (Fig. 1D and equations 3, 4)). These steps result in a wave similarity map such as shown in Fig. 1E. The application of OWS during arrhythmia (ventricular fibrillation, VF) is shown in Supplementary Fig. S1. The calculation of the regularity index (RI) follows the same processing steps of windowing, normalisation and alignment. However, for calculation of RI the wave distance is instead calculated (equation (5)) and collapsed to 0 or 1 based on a defined distance threshold (equation (6)). The relationship between these two measures is explored below.

### 3.2 Wave similarity detects temporal heterogeneity in computationally modelled and optically recorded action potentials

**Fig. 2:**
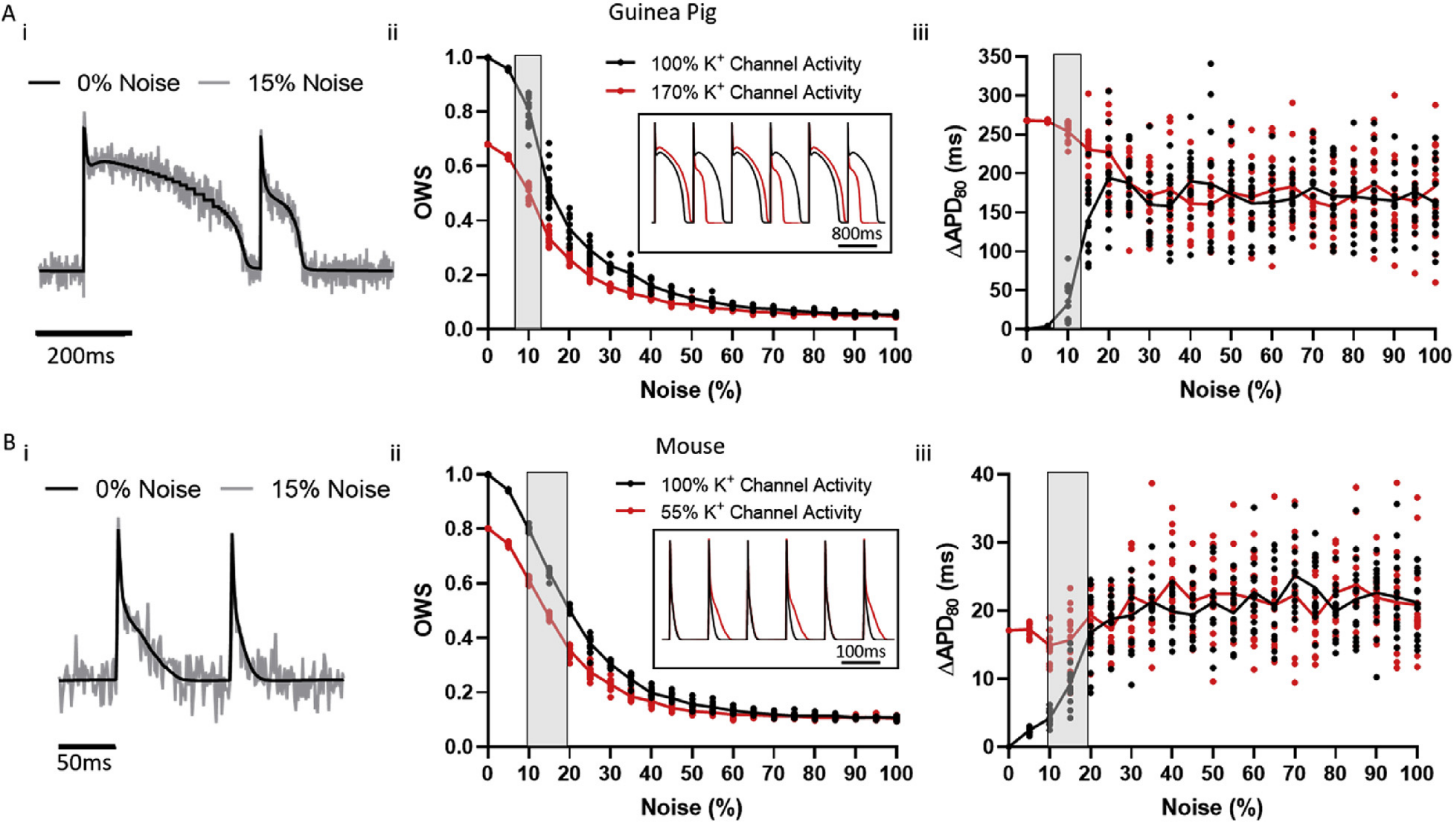
Validation of the use of optical wave similarity (OWS) for measurement of modelled alternans behaviour. Ai) Example modelled guinea pig action potentials without (black) noise and with addition of 15% noise (grey). ii) OWS measurement of computationally modelled guinea pig action potentials without (black) and with (red) temporal irregularity as a function of noise. Inset shows the two modelled, normalised, signals used (with no noise). iii) APD_80_ alternans (*ΔAPD*_*80*_) of computationally modelled guinea pig action potentials without (black) and with (red) temporal irregularity as a function of noise. Grey areas show the noise range observed in our experimental optical recordings from guinea pig whole hearts. Bi) Example modelled mouse action potentials with (15%, grey) and without (black) noise addition. ii) OWS measurement of computationally modelled mouse action potentials without (black) and with (red) temporal irregularity as a function of noise. Inset shows the two modelled, normalised, signals used (with no noise). iii) *ΔAPD*_*80*_ of computationally modelled mouse action potentials without (black) and with (red) temporal irregularity as a function of noise. Grey areas show the noise range observed in our experimental optical recordings from mouse atria.

To initially validate whether this approach could be utilised to quantify temporal regularity, we measured OWS in model action potentials. Guinea pig and mouse model action potentials were utilised, with and without temporal alterations. Temporal alterations replicating alternans (Fig. 2A and B) were generated by altering the conductance of the respective repolarising potassium channels in each model for odd beats only, as described in the methods. In the absence of noise, OWS was reduced by the presence of alternans. For guinea pig action potentials, alternans reduced OWS from 1 to 0.68 (Fig. 2Aii), while mouse alternans reduced OWS from 1 to 0.80 (Fig. 2Bii). These differences in measured OWS values are maintained as noise levels are increased to those expected in experimental setups (noise of 8–20% as observed in our experimental work, highlighted in grey in Fig. 2A and B). As noise is increased beyond these levels however, both the temporally regular and irregular signals exhibit similar and low OWS values, reducing to circa 0.050 and 0.11 for the guinea pig and mouse respectively.

APD alternans magnitude (*ΔAPD*_*80*_) is increased in the model data without noise, from 0 ms to 268 ms and 17.1 ms for guinea pig and mouse action potentials respectively, Fig. 2Aiii and Biii. Increased *ΔAPD*_*80*_ in the temporally irregular signals is maintained at experimental noise levels. As noise is increased beyond these experimental values, it is no longer possible to detect the differences in APD alternans magnitude between the temporally regular and irregular signals. This convergence occurs quicker than for OWS, suggesting improved sensitivity for alternans detection compared to standard alternans quantification method. For example, at 25% noise level OWS is still significantly lower in the guinea pig temporally regular signal (0.29 ± 0.01 vs 0.20 ± 0.004, no alternans vs alternans, P < 0.0001). For *ΔAPD*_*80*_ however, there is no significant difference (187.4 ± 8.4 ms vs 187.5 ± 9.7 ms, no alternans vs alternans, P = 0.99). Similarly, for mouse action potentials at 25% noise OWS is still lower (0.38 ± 0.01 vs 0.28 ± 0.01, no alternans vs alternans, P < 0.0001), but there is no difference in *ΔAPD*_*80*_ amplitude (18.8±1 ms vs 17.2 ± 0.9 ms, no alternans vs alternans, P = 0.26).

**Fig. 3:**
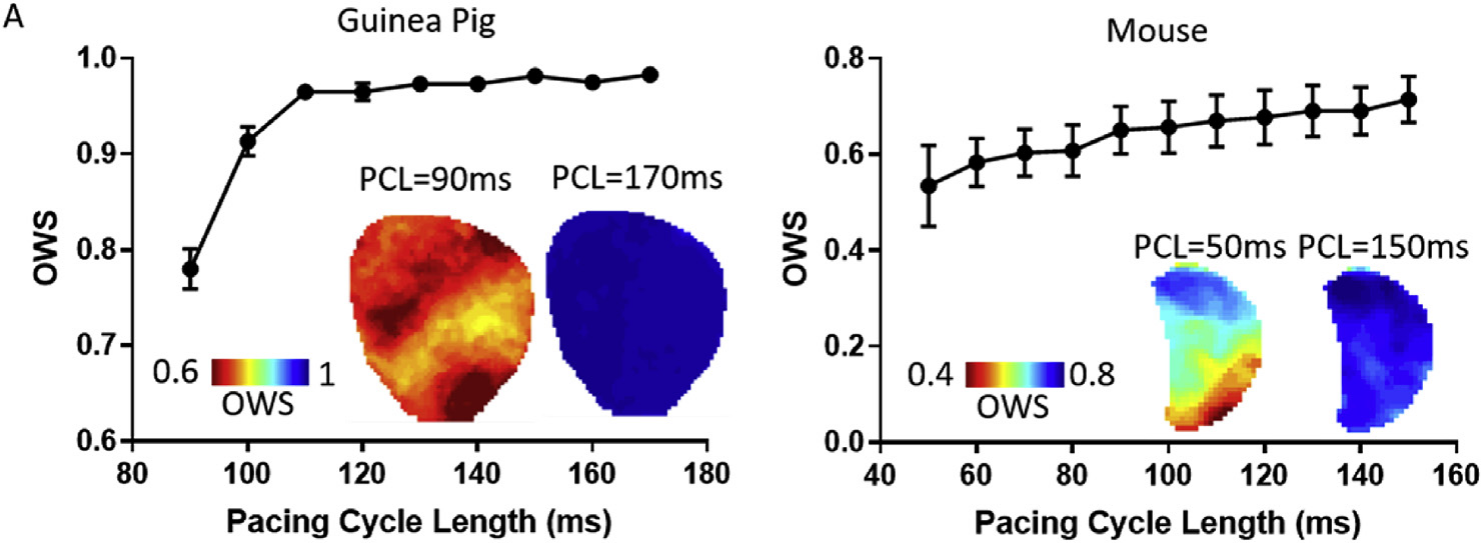
Validation of the use of optical wave similarity (OWS) for measuring action potential regularity. A) OWS as a function of pacing cycle length (PCL) in optically mapped guinea pig hearts, n = 6. Inset shows example OWS maps at PCL = 170 ms and 90 ms respectively. B) OWS as a function of PCL in optically mapped mouse left atria, n = 7. Inset shows example OWS maps at PCL = 150 ms and 50 ms respectively.

Dynamic decreases in PCL are known to induce greater temporal action potential variability due to the occurrence of AP alternans (Johnson et al., 2010). As an initial investigation into the use of OWS analysis in optically recorded action potentials, we therefore quantified OWS at progressively shorter PCLs in guinea pig whole hearts and mouse atria, Fig. 3. In both experimental models, OWS decreases with decreasing PCL as expected, Fig. 3A and B. OWS analysis therefore is an effective measure of temporal homogeneity in optically mapped datasets. To test the sensitivity of OWS measure to window setting, we repeated the analysis of OWS as a function of PCL in the guinea pig heart with altering window lengths (50–1000 ms). In all cases, OWS reduced with decreasing PCL, Supplementary Fig. S2A.

### 3.3 Correlation of wave similarity and regularity index measures

**Fig. 4:**
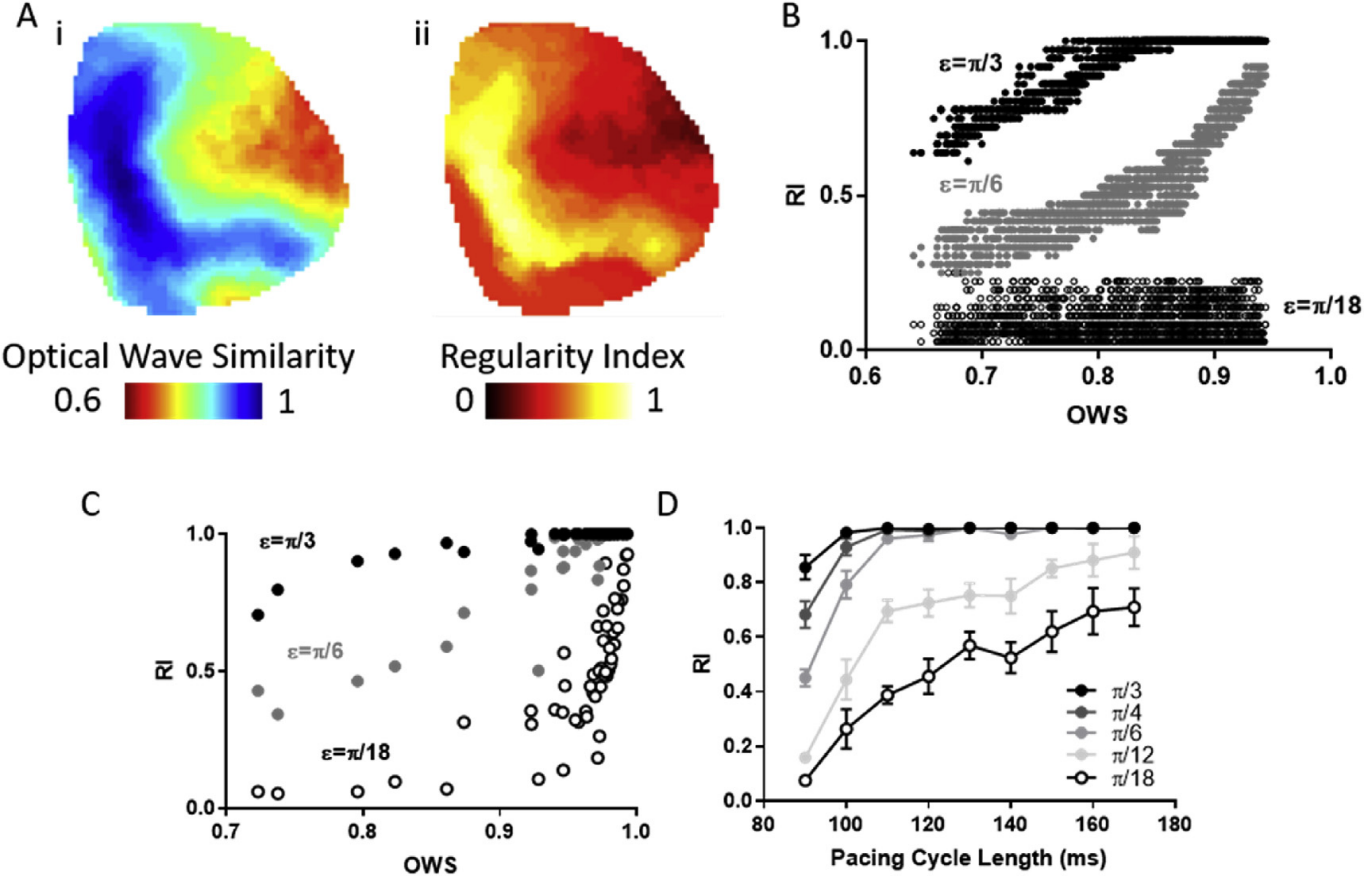
Correlation of regularity index (RI) and optical wave similarity (OWS) measures in guinea pig whole hearts. A) Example OWS (i) and RI maps (ii, ε = π/6) at PCL = 90 ms. B) Spatial correlation between OWS and RI measures in the guinea pig heart (r^2^ = 0.86). C) Correlation of mean OWS and RI measures in guinea pig whole hearts across PCLs. D) RI measure as a function of distance threshold (ε). n = 6 hearts, 9 PCLs per heart.

The measurement of OWS and the electrogram derived regularity index (RI) (Faes et al., 2002) is set out by equations 4, (6)) respectively. To test the correlation between these two methods, RI analysis was applied to guinea pig whole hearts at different distance thresholds (ε). Fig. 4A and B demonstrate that, as expected, areas of low OWS in an individual heart concurrently demonstrate low RI when an appropriate threshold value is set (e.g. when ε = π/6, r^2^ = 0.79). However, when a small distance (high similarity) threshold is set, correlation between the measures is lost (e.g. when ε = π/18, r^2^ = 0.012). Furthermore, setting a large distance threshold such as ε = π/3 is observed to reduce the dynamic range of the RI measure, as many areas are measured to have RI = 1 when the OWS measure suggests there are differences in temporal homogeneity (OWS values ranging from 0.77 to 0.94). Setting of the threshold value is also observed to affect mean RI measurements across whole hearts at various PCLs, Fig. 4C. Again, setting a large distance threshold of π/3 leads to many samples of RI = 1, reducing dynamic range. A high similarity, low distance, threshold of π/18 reduces correlation with OWS measure, (r^2^ = 0.51 compared to r^2^ = 0.86 when ε = π/6).

These results demonstrate that a key aspect of RI analysis is setting of an appropriate threshold value, ε. We therefore measured RI in guinea pig whole hearts as a function of changing threshold as shown in Fig. 4D. As expected, and proportional to the OWS response (Fig. 3C), RI decreased with decreasing PCL. RI therefore, like OWS, appears to be an effective measure of temporal homogeneity in optically mapped datasets. However, considering that the choice of ε dramatically alters ‘baseline’ values and response to decreasing PCL we opted to utilise OWS in subsequent studies.

### 3.4 Beat-to-beat wave similarity detects short periods of temporal instability

**Fig. 5:**
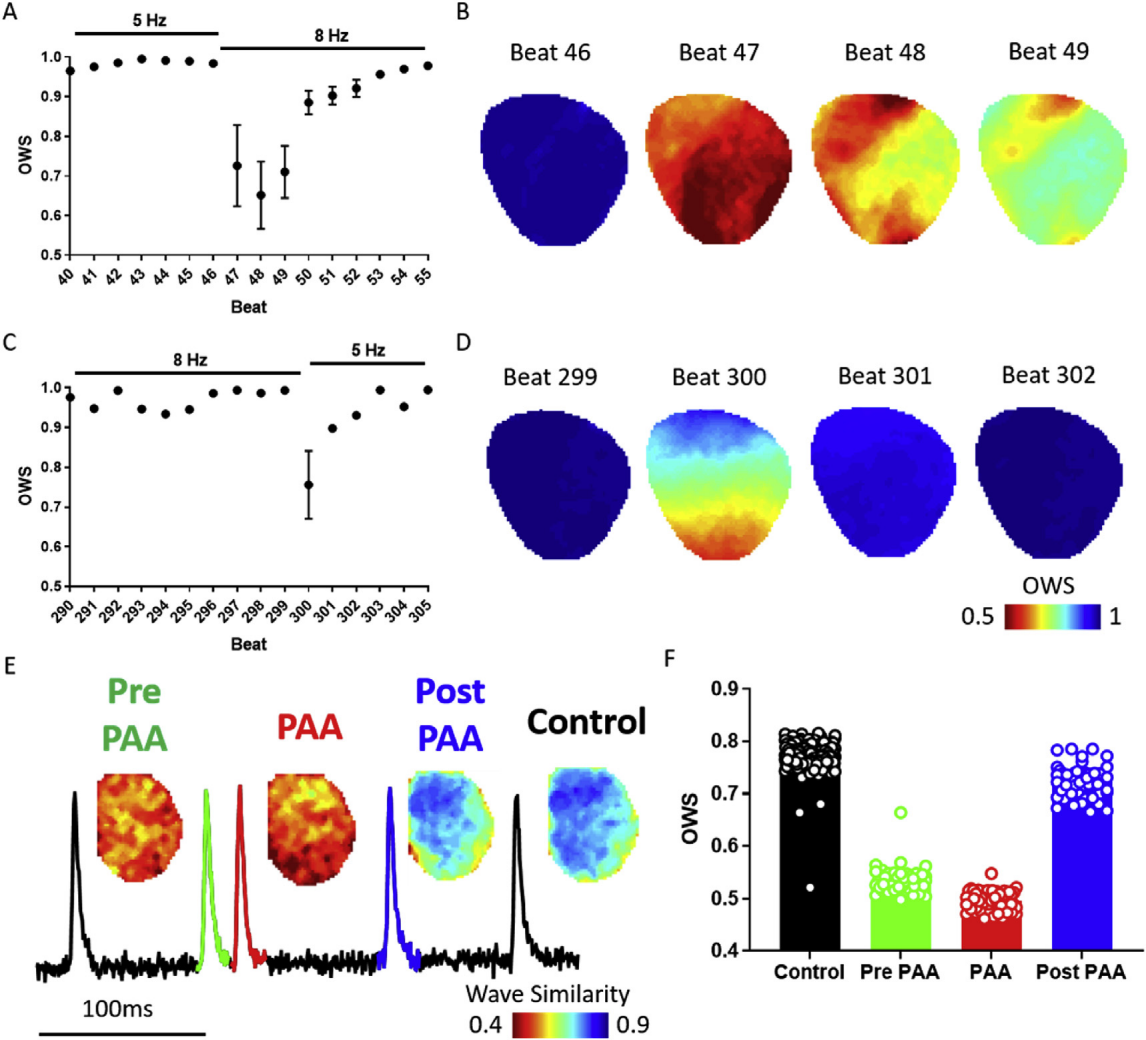
Single beat optical wave similarity mapping. A) Single beat OWS as a guinea pig heart transitions from 5 Hz to 8 Hz pacing. B) OWS maps immediately before (Beat 46) and after (Beat 47–49) pacing frequency change. C) Single beat OWS as the heart transitions from 8 Hz to 5 Hz pacing. D) OWS maps immediately before (Beat 299) and after (Beat 300–302) pacing frequency change. E) Example trace of a premature atrial activity (PAA, red) and example OWS maps. F) Mean data of OWS for all beats in the experiment classified as control (black), pre-PAA (green), PAA (red) and post-PAA (blue).

Acute variations in pacing cycle length are known to induce temporal instability in the electrical behaviour of the heart (O’Shea et al., 2019a). To test OWS analysis in this context, we utilised this parameter to analyse beat-to-beat heterogeneity in a guinea pig whole heart which underwent a sudden increase in pacing frequency from 5 Hz to 8Hz and then returned to 5Hz, Fig. 5A–D. OWS analysis identified a transient period of temporal heterogeneity immediately after the onset of 8 Hz pacing (Fig. 5A and B). Similarly, return to 5 Hz pacing induces a reduction in OWS from 0.99 to 0.75, but again the temporal stability of the heart recovers quickly, Fig. 5C and D. Interestingly, immediately after the cycle length change a substantive heterogeneity in OWS is observed between the apex and the base of the heart. Follow up studies will address the pathophysiological significance of this regional heterogeneity.

We also investigated how OWS performs in the setting of spontaneous electrical premature atrial activity (PAA). Mouse left atrium was exposed to 150 ms PCL and areas of the trace exhibiting PAAs were analysed, Fig. 5E. The morphology of the OAPs of the PAAs is seen to be markedly distinct, with OWS = 0.49 ± 0.02 and 0.53 ± 0.02 when compared to the APs immediately before and after the PAA. In contrast, OWS = 0.73 ± 0.03 for APs following the PAA, and 0.77 ± 0.02 for all other ‘control’ beats, Fig. 5E and F. These results are collaborated by traditional action potential duration (APD) analysis. APD70 of the ‘control’ beats in this study was 13.3 ± 0.02, vs 12.9 ± 0.07 for the PAAs for example. OWS mapping therefore suggests that the PAAs are associated with potentially proarrhythmic periods of temporal instability.

### 3.5 Optical wave similarity detects and quantifies alternans and predicts ventricular fibrillation onset

**Fig. 6:**
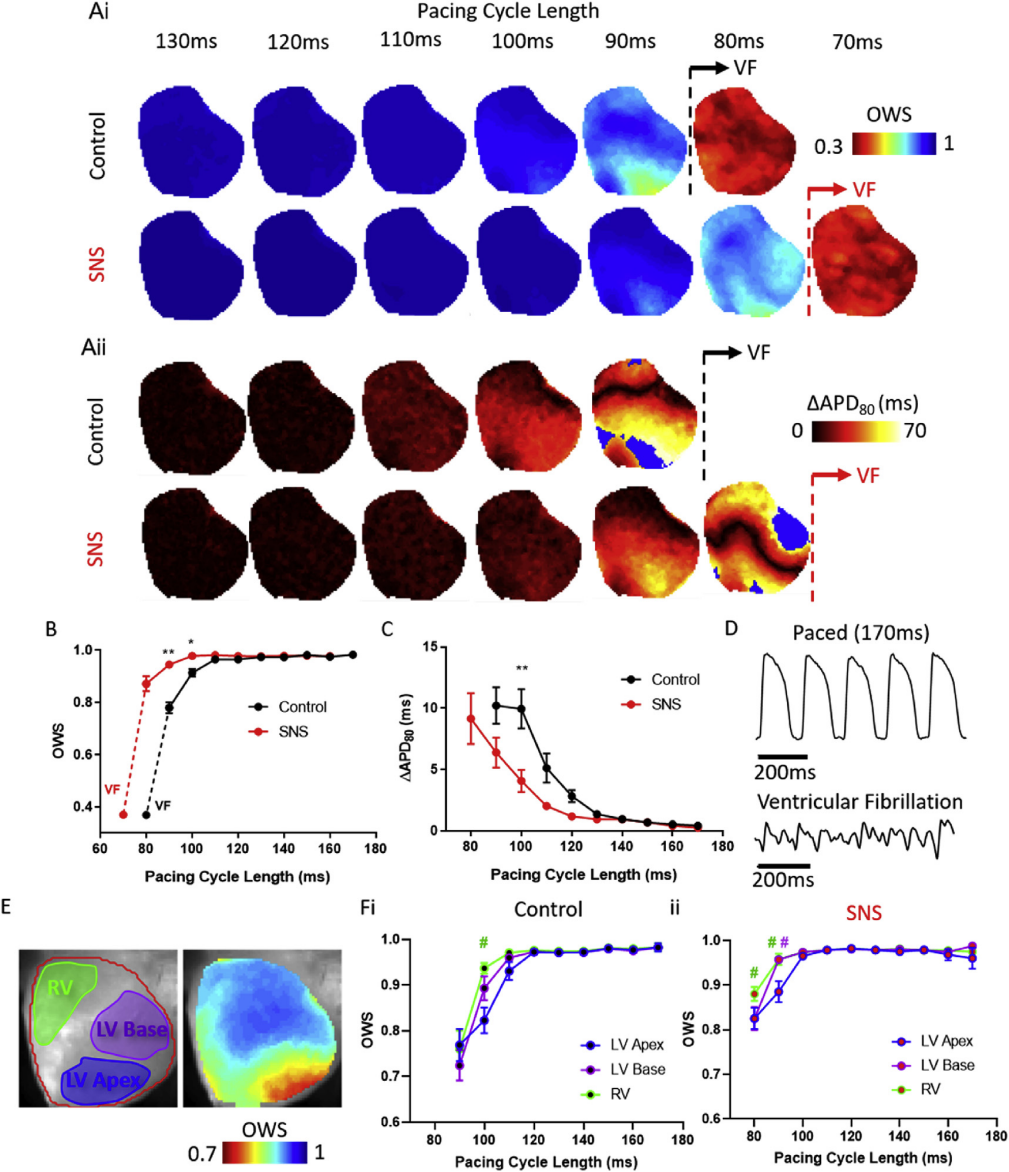
Effects of sympathetic nervous stimulation on optical wave similarity (OWS) and APD alternans (*ΔAPD*_*80*_) measures. A) Example OWS (i) and APD_80_ alternans (ii) maps from 130 to 70 ms PCL in a guinea pig heart without (control, black) and with SNS (red). The transition to ventricular fibrillation (VF) is marked by the dashed line in both conditions. Blue areas in APD alternans maps denote areas not analysed due to 80% repolarisation not being reached. B) Grouped data of mean OWS values as a function of PCL in control hearts with/without SNS, before (solid line) and during (dashed line) VF. C) Grouped data of mean *ΔAPD*_*80*_ as a function of PCL in control hearts before and during SNS, before onset of VF. D) Example single pixel optical traces recorded during 170 ms PCL stimulation and during VF induced via pacing. E) Anatomical locations of left ventricle (LV) Apex (blue), base (purple) and right ventricle (RV, green) on fluorescence image of voltage dye loaded guinea pig whole heart used for regional studies of OWS. F) Grouped data of regional OWS values as a function of PCL in control hearts with (i) and without (ii) SNS. n = 6 hearts, *P < 0.05, **P < 0.01 control vs SNS, #P < 0.05 LV Apex vs RV (green) or LV Base (purple).

Dynamic pacing at shorter PCLs induces alternans thereby increasing temporal heterogeneity. Previous work has demonstrated that the PCL required to initiate VF is shorter during SNS of intact guinea pig hearts, showing that SNS is protective against the onset of ventricular fibrillation by the suppression of alternans (Winter et al., 2018). To test how the OWS analysis compares to established measures of beat to beat heterogeneity, namely APD alternans, OWS and alternans magnitude were quantified in guinea pig hearts loaded with a voltage-sensitive dye during SNS and control (no SNS) conditions (preceding onset of VF), Fig. 6A–C. As previously shown, APD alternans magnitude at increased PCL was shortened (Fig. 6Aii and C). Control hearts showed a significantly increased alternans amplitude compared to SNS at PCL of 100 ms (*ΔAPD*_*80*_ = 9.95 ± 1.6 ms vs 4.07 ± 0.9 ms, control vs SNS, P = 0.0078). OWS analysis showed comparable changes in temporal heterogeneity. As PCL is decreased, OWS decreases (Fig. 6Ai and B) and at PCL of 100 ms OWS = 0.91 ± 0.02 vs 0.98 ± 0.02 (control vs SNS, P = 0.0106). Similar results were seen at all but the longest window settings tested, Supplementary Fig. S2B. There were also regional differences in the onset of temporal heterogeneity, Fig. 6E–F. In control conditions at PCL = 90 ms, the left ventricular (LV) apex is observed to exhibit low OWS when compared to the right ventricle (RV), OWS = 0.82 ± 0.03 vs 0.94 ± 0.01, LV apex vs RV, P = 0.045). In SNS at PCL = 90 ms, the LV apex (OWS = 0.89 ± 0.02) is again observed to exhibit lower OWS versus both the RV (0.96 ± 0.01, P = 0.0317) and LV base (0.96 ± 0.01, P = 0.0362). Therefore, OWS effectively distinguishes between physiological interventions that affect temporal homogeneity in optically recorded action potentials, demonstrating here that hearts without SNS are less temporally stable at the same PCLs. OWS also highlights regional variations in temporal stability, which may have arrhythmogenic consequences.

The hearts without SNS (control) enter VF at slower PCLs (Fig. 6) (Winter et al., 2018), demonstrating the use of OWS in a similar manner as APD alternans as a metric for arrhythmia inducibility. The rapid and disorganised OAPs recorded in arrhythmic data such as during VF makes traditional parameter measurements (APD, alternans, cycle lengths etc) difficult to perform (Kirchhof et al., 1998), Fig. 6D. We therefore tested whether OWS analysis could be used to quantify data during VF. Indeed, OWS is greatly decreased during VF, Fig. 6B, suggestive of large temporal heterogeneity in OAPs recorded during arrhythmia. OWS during VF was 0.37 ± 0.02 and 0.37 ± 0.01 for control and SNS respectively, and there was no difference between conditions (P = 0.90).

We also mapped dominant frequency in the hearts during VF. No correlation was found between the two measures (Supplementary Figs. S3A–B). Dominant frequency during VF was reduced in control hearts compared to those with SNS (14.4 ± 0.3 vs Hz 16.4 ± 0.5 Hz, P = 0.0085, Supplementary Fig. S3C).

## 4 Discussion

Here, we have developed and tested the application of OWS mapping as an approach for analysis of pro-arrhythmic and arrhythmic activity in cardiac optical mapping datasets, Fig. 1. This approach has been inspired primarily from the development of ‘regularity index’ measures, originally designed for use in atrial electrograms during AF (Faes et al., 2002; Ravelli et al., 2005). Arguably, however, the results shown here demonstrate that the most promising use of OWS analysis in optical datasets may be mapping temporal stability of OAPs before the occurrence of arrhythmias, thus providing a novel index identifying period of electrical instability directly preceding arrhythmias. In Fig. 6 for example, SNS stimulation is shown to supress temporal heterogeneity induced by decreasing PCL, Fig. 6B. These results align with alternans analysis of the datasets with the same sensitivity, with a significant difference between the control and SNS groups evident with both measures at PCL = 100 ms, Fig. 6B and C (Winter et al., 2018). Measuring APD alternans however requires the measurement of baseline signal level, activation time (for which several different methods are proposed (O’Shea et al., 2019a)), peak time and repolarisation time from the OAPs. As shown in Fig. 2, noise can therefore corrupt APD alternans measures, and restrictive definitions on activation and repolarisation times can prevent analysis of APD, Fig. 6Aii. In contrast, OWS analysis foregoes the need for quantification of these specific and inconsistently defined signal features/points (Laughner et al., 2012).

Furthermore, misidentification of these parameters may lead to false positives or false negatives and may explain the loss of significant difference between the two conditions at PCL = 90 ms, which is evident with OWS analysis. OWS analysis therefore provides a simpler and more easily reproducible processing and analysis pipeline that incorporates entire OAP signal morphology alternations. Additionally, OWS measurement, like alternans analysis, can be performed on a beat-to-beat basis to highlight acute periods of instability, Fig. 5. However, in contrast to alternans analysis, OWS is not designed to study a specific form of temporal instability, and hence can be applied to analyse nonperiodic alterations in cardiac electrophysiology, such as apparently random alterations in beat-to-beat variability that are observed in varied proarrhythmic conditions (Johnson et al., 2010).

OWS, as expected, is proportional to RI mapping results as shown in Fig. 4. RI analysis has been utilised in several important studies of temporal regularity including deciphering the difference in electrical behaviour during paroxysmal and chronic AF (Ravelli et al., 2005). Fig. 4D however shows that the selection of the threshold value ε impacts greatly on the measured baseline RI values (at slowest PCL), and the response to decreased PCL. OWS analysis did not involve the setting of such a cut off value, so was deemed more appropriate for these initial studies into the effectiveness of regularity-based measures in optical mapping. Furthermore, the dynamic range of measured OWS values can be reduced by threshold selection. For example, many samples demonstrate mean RI values of 1 when ε = π/3, despite exhibiting a range of OWS values (0.77–0.94), Fig. 4C. RI mapping therefore may result in subtle changes in waveform morphology temporal stability, for example with small changes in PCL, not being identified.

During arrhythmia (in this case VF), OWS detects an increase in temporal heterogeneity, Fig. 6B and D. This demonstrates that it can be used as a simple marker for arrhythmia onset. However, further studies are required to extend its use to classify sub-types of arrhythmia or arrhythmia severity in optical mapping data. The same alignment and windowing strategy as used for the non-arrhythmic (minima before and after the alignment time) was used to analyse the hearts during VF. Furthermore, during VF the use of equation (1) to detect peaks and hence define alignment times is potentially flawed due to low signal amplitude and regional heterogeneity in activation rates. An important future study therefore would be optimisation of identifying active times for arrhythmic data (Hajimolahoseini et al., 2018).

Another important consideration is how much lower OWS values report noise in the signal compared to physiological variations. From the model studies conducted, it is observed that OWS decreases with increased noise, and asymptotically approaches a low but non-zero value as noise is increased to levels far beyond those we saw in our experimental recordings, where it can be assumed that any underlying regularity is completely masked by noise. The values of OWS observed in the extreme noise model signals (circa 0.05 and 0.11 for guinea pig and mouse respectively) are much less than the values of OWS measured during VF (0.37 ± 0.02), signifying that there is still regularity in the signals. This suggests that, although noise clearly impacts OWS analysis (Fig. 2A and B), it could still be utilised as a method of sub-classifying arrhythmias based on temporal stability. Further studies looking into distinct arrhythmia types are required to confirm this hypothesis, as in the present study only VF was studied, with and without SNS. Analysis resulted in the same values of OWS being recorded in both conditions, suggesting no difference in these arrhythmias in terms of temporal stability (Fig. 6B).

OWS provides a single metric designed to report temporal stability. Analysis of electrogram recordings however suggest that value is gained by utilising these measures concurrently with other measures to identify critical sources or substrates. These measures can combine OWS analysis with other time domain (Baher et al., 2019) (e.g. cycle length by barycentre estimation (Ravelli and Mase, 2014)) or frequency domain metrics (Chang et al., 2013). Therefore, rate and regularity are combined, and it has previously been shown how drivers of AF can exhibit rapid (i.e. high dominant frequency) but regular (high OWS) electrical impulses (Warren et al., 2006). The fact that (i) no correlation was observed between dominant frequency and OWS measures (Supplementary Figs. S3A–B) and (ii) dominant frequency analysis distinguished between control and SNS conditions when OWS does not (Fig. 6E and Supplementary Fig. S3C) supports the idea that these two measures contain divergent information about the electrical behaviour of cardiac tissue. It is suggested that a combined approach, in electrogram mapping, allows classification of sites not just simply by temporal regularity, but also into drivers/sources of arrhythmia and arrhythmia substrates (Ravelli and Mase, 2014). The application of such an approach for optical data therefore warrants further investigation.

As highlighted, a potential advantage of OWS over other methods is the more limited number of signal features that need to be identified, increasing ability for automated analysis. However, optical mapping experiments are conducted in several experimental models with distinct AP morphologies. Furthermore, alongside OAPs, cytosolic and sarcoplasmic reticulum calcium concentrations can be optically measured with calcium sensitive dyes (Wang et al., 2014; Jaimes et al., 2016). The recorded optical calcium transients will also exhibit a distinct wave morphology compared to OAPs. Therefore, further validation and optimisation of OWS is required with different models and fluorescent dyes. In these studies, OWS is shown to be slightly dependent on window size, although only very long window size is shown to effect results of decreasing PCL or applying SNS, Supplementary Fig. S2. More automated windowing methods, including using time or frequency domain based measured features, may improve the method.

A key aspect of this work is the incorporation of these techniques into our freely available mapping software ElectroMap (www.github/CXO531/ElectroMap) (O’Shea et al., 2019a). From ElectroMaps updated user interface, OWS and RI mapping can be applied with user defined alignment strategy, distance threshold and windowing options, broadening the application of these analyses to different experimental models and fluorescent sensors not presently tested.

## 5 Conclusion

OWS mapping provides effective measures of temporal regularity that can be applied in optical datasets. Using these methods, physiological alternations down to single beat timescales can be quantified, and different states can be classified which are known to suppress arrhythmia. By considering the whole signal morphologies of OAPs, the definition of specific signal features is not required, and alterations in any part of the AP will contribute to the local OWS measure.
