# Supplementary material for: Temporal irregularity quantification and mapping of optical action potentials using wave morphology similarity
Source: Prog Biophys Mol Biol. 2020 Nov;157:84–93. doi: 10.1016/j.pbiomolbio.2019.12.004 (PMC7607254; doi:10.1016/j.pbiomolbio.2019.12.004)
Supplement: Multimedia component 1 [file mmc1.docx]

**Supplementary Information**

**
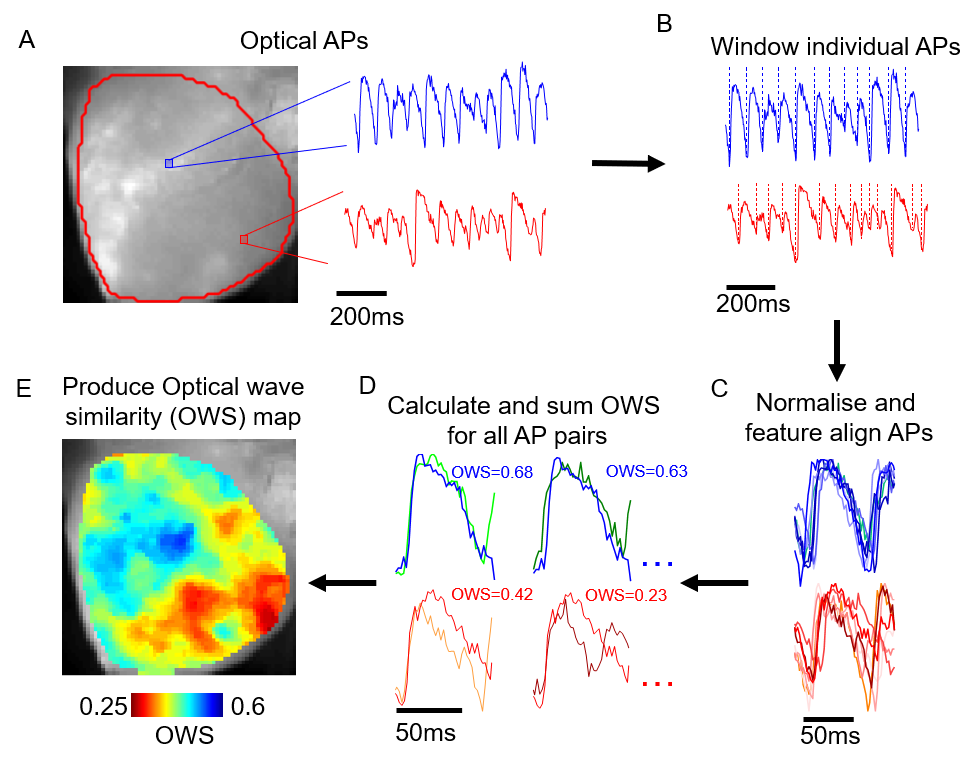
**

**Supplementary Figure S1: Calculation of optical wave similarity (OWS) from voltage optical mapping data from a heart during ventricular fibrillation (VF). A)** Fluorescence image of voltage dye loaded guinea pig whole heart. Representative signals are shown from the locations marked in blue and red on the fluorescence image. **B)** Windowing of optical signals based on signal minima. **C)** Normalisation and alignment of individual optical action potentials (OAPs) in the recorded optical signal. **D)** Example calculations of OWS from OAP pairs. **E)** Constructed map of OWS following analysis steps set out in A-D.


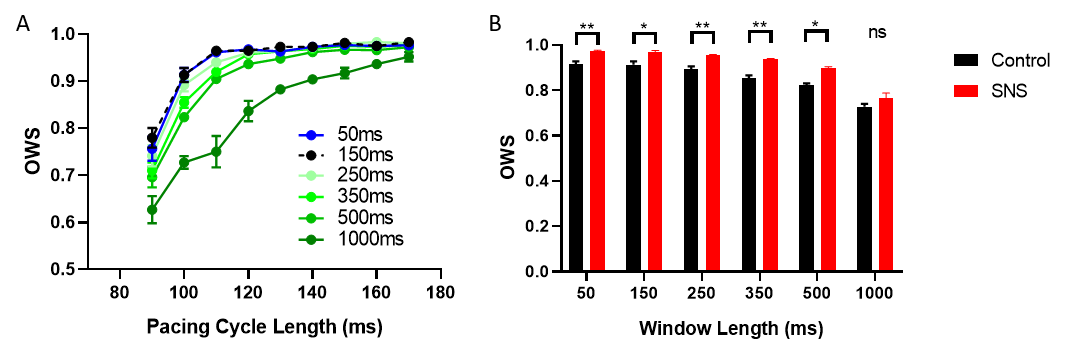


**Supplementary Figure S2: Optical wave similarity (OWS) measure as a function of window size in guinea pig whole hearts. Window length refers to time after peak, and time before peak was held at 50ms. A)** OWS as a function of pacing cycle length for different window sizes from 50ms (blue) to 1000ms (green). **B)** OWS at different window lengths with (red) and without (black) sympathetic nervous stimulation (SNS) at 100ms PCL. n=6 hearts, *p<0.05, **p<0.01 control vs SNS


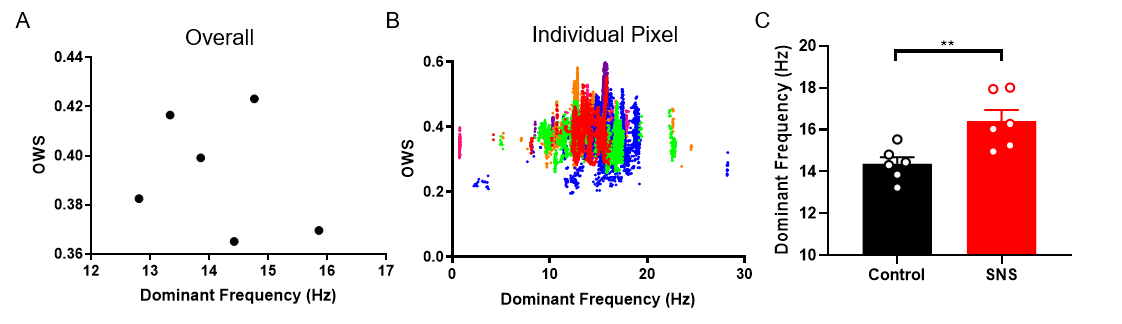


**Supplementary Figure S3: Correlation between optical wave similarity (OWS) and dominant frequency measures during ventricular fibrillation (VF). A)** Mean OWS and dominant frequency values of each heart**. B)** Correlation between OWS and dominant frequency values. Each colour represents a different heart, while each point represents a different pixel. **C)** Dominant frequency with (red) and without (black) sympathetic nervous stimulation in hearts during VF. n=6 hearts, **p<0.01 control vs SNS
